# Basic Income in Belgium survey: experimental data on citizens’ attitudes toward a variety of basic income policies

**DOI:** 10.1016/j.dib.2023.109376

**Published:** 2023-07-07

**Authors:** Tijs Laenen, Marie-Laure Mulayi, Cyrille Francisco, Wim Van Lancker

**Affiliations:** Centre for Sociological Research, KU Leuven, Parkstraat 45, 3000 Leuven, Belgium

**Keywords:** Vignette experiment, Opinion survey, Basic income, Welfare attitude, Multidimensionality

## Abstract

This article presents the BAsic income in BELgium (BABEL) dataset on public opinion on the introduction of a universal basic income (UBI) in Belgium, collected through an online panel among a sample of 3000 respondents in spring 2021. The BABEL survey implements an innovative vignette experiment in which both the policy design (i.e., the benefit level, the universality) and the potential policy outcomes (i.e., effect on poverty, unemployment) of a UBI are set to vary randomly. This full factorial experimental design is appropriate to analyze the complex of process of opinion formation about a UBI which entails multiple considerations. Accordingly, the data enables researchers to assess the net effect of the different design characteristics and hypothetical outcomes, as well as the trade-offs people are (not) willing to make to support basic income. Additionally, the survey includes items about benefit recipiency, COVID-19, demographic characteristics, general welfare attitudes, behavioral intentions, and political opinion. These data are thus appropriate for examining which design or outcome factors are relevant in shaping support for a UBI as well as extensive subgroup analysis.

**Specifications Table**

**Table:** 

Subject	Social sciences; Welfare attitudes; Public opinion
Specific subject area	The specific area of research is public support for a universal basic income.
Type of data	Survey results in tables
How the data were acquired	Through an online survey in Qualtrics. The questionnaire is included in this article.
Data format	Processed and raw data (cleaned and vignette variables are formatted).
Description of data collection	The 3000 survey respondents were recruited via an opt-in panel of the largest Belgian online panel, Bilendi To limit self-selection bias, the agency only informed its members about the subject of the survey after they agreed to participate. The general participation rate was about 25%, meaning that one in four of those who were invited to participate in the survey actually did so. The quality of the survey answers was monitored (e.g. by checking the time needed to fill in a survey and the consistency of the answers) and excludes those who provide low-quality data. The sample is already representative of the Belgian population in terms of age, gender and language. Nevertheless, the data file does contain a weighting factor based on these characteristics, which also includes education. Important to note is that because education was not used as an interlaced variable in the sampling, there is a larger (but still very small) deviation between the sample and the population distribution. The weighting factor corrects for under- or overrepresentation of certain groups by dividing the number of people to be achieved in the group according to the population distribution by the number of people to be achieved in the group in the sample. Respondents with a weighting higher than 1 belong to a group that is underrepresented in the sample compared to the population. Respondents with a weighting factor lower than 1 belong to a group that is overrepresented in the sample compared to the population.
Data source location	Leuven, Belgium
Data accessibility	Repository name: OSF Data identification number: https://doi.org/10.17605/OSF.IO/7GHFS
Related research article	Not applicable.

## Value of the Data

• The dataset provides valuable information on how different policy design dimensions (e.g., the institutional embeddedness, universality, conditionality, amount and financing) and policy outcomes dimensions (e.g., effect on income inequality and entrepreneurship, informal care, unemployment and poverty) affects support for a UBI. With this information, researchers can thoroughly analyze the net effect of each design and outcome characteristic on respondents' evaluation of a UBI, as well as assess the feedback effects of a policy that does not (yet) exist.
• These data contain unique information about the possible behavioral consequences of a UBI, or in other words, insights into what people would actually do if they received a monthly allowance from the government. Respondents could choose from several non-exhaustive options such as quitting work, working less, volunteering, starting their own business, etc. This question provides a unique opportunity to examine possible substitution effects among different subgroups, i.e. women, benefit recipients, resulting from the implementation of a UBI.
• Additionally, these vignette dimensions also can be interacted with one another which enables the assessment of the trade-offs people are (not) willing to make to support a UBI as well as the relative importance of each vignette dimension. For instance, the universality dimension (including newcomers - residency requirements - nationality requirements) can be interacted with the conditionality dimension (unconditional – conditional) in order to assess what conditions are imposed on which population to gain access to a basic income.
• The dataset also includes items on political beliefs, general welfare attitudes, Covid-19, socio-demographic information and attitudes towards other similar policy proposals (such as a negative income tax and a basic endowment). This information enables thorough subgroup analyses and other related theory-driven research about which mechanisms (self-interest, ideological inclinations, etc.) could drive support for a UBI.
• These data includes several qualitative questions (i.e., how do you define a UBI) that allow a fine-grained investigation of respondents’ subjective understanding of the proposal which is lacking in existing studies.

## 1 Objective

The main objective of the BABEL Survey is to contribute to the limited knowledge about the multidimensionality of popular support for a UBI [1]. First, we are among the first to establish a causal link between the institutional design of a UBI and its social legitimacy. For that purpose, we rely on unique vignette experiments in which respondents rate hypothetical a UBI scenarios that randomly vary on multiple policy design dimensions. Because they are gathered among large, representative population samples, such vignette (or factorial) surveys bring together the best of two methodological worlds: the internal validity and causal inference of the experiment, and the external validity and generalizability of the survey (Auspurg & Hinz, 2015). Second, we analyze a much broader variety of UBI types, by expanding the number of policy design dimensions (as well as the number of options within these dimensions) in our experiments. Based on existing welfare state literature, we argue that a UBI can be assembled with the same eight ‘building blocks’ as any other income benefit: access, administration, conditionality, duration, financing, level and position within the larger welfare system. Within these dimensions, numerous policy options are available, leading to a plethora of possible BI varieties. The vignette approach adopted in the current research project allows us to examine popular support for a much larger sample of BI types than would be possible with traditional surveys.

## 2 Data Description

Table 1 shows the population distribution, sample distribution and participation rate per language, age, gender and education. As previously stated, the sample is representative of the Belgian population as a result of the sampling procedure. Table 2 and 3 respectively show the dimensions, levels and wording for the vignette experiment on policy design and outcome.

Table 4 presents the average level of support for a UBI by randomized levels for the policy design and outcomes. First, we note that Belgian citizens are more in favor of a conditional (i.e., required to care or volunteer) and generous (i.e.,1500 €) a UBI that imposes specific eligibility requirements (i.e., nationality or residency). They also prefer a UBI that complements existing social benefits and is financed by a capital or climate taxation. Second, the opinions about the hypothetical outcomes show that the level of support increases for a UBI that would tackle social problems (i.e., unemployment or income inequality). Moreover, there is clear evidence that a UBI that would both stimulate entrepreneurship or increase the number of care givers receives the most public support.

**Table 1:** Population distribution, sample distribution and participation rate per group.

Language	Age	Gender	Sample distribution
Dutch	18-34 35-54 55+	men women men women men women	7.6% 7.5% 10.0% 9.8% 10.3% 10.7%

French	18-34 35-54 55+	men women men women men women	6.6% 6.6% 8.0% 7.9% 7.1% 7.9%

**Education**			

Secondary at most Tertiary (i.e. Bachelor or Master)			57.7% 42.3%

**Table 2:** Dimensions, levels and wording for the first vignette experiment on policy design.

Dimensions	Levels	Vignette text
Amount	500	The Belgian government provides a monthly income of 500€.
	1000	The Belgian government provides a monthly income of 1000€.
	1500	The Belgian government provides a monthly income of 1500€.
Universality	Fully universal	That amount is paid to all adult residents in Belgium, including newcoming migrants.
	Residency requirement	That amount is paid to all adults living in Belgium, on the condition that they been in the country for a few years.
	Nationality requirement	That amount is paid to all adults living in Belgium, on the condition that they have the Belgian nationality.
Conditionality	Unconditional	People who are not working are not obliged to search for a paid job.
	Conditional on participation	People who are not working are obliged to do voluntary work or to take up caring responsibilities.
Integration	Replacement: all	The basic income replaces all existing social benefits.
	Replacement: some	The basic income replaces some existing social benefits, such as child allowances and sickness benefits.
	Replacement: some but top-ups	The basic income replaces some existing social benefits but provides top-ups for people with additional needs or costs (e.g. people with children or disabled people).
	Replacement: none	The basic income replaces no existing social benefits.
Financing	Climate tax	The basic income is paid for by income taxes and social security contribution, which stay as they are. Potential additional costs are covered by a new climate tax on CO^2^ emissions.
	Capital tax	The basic income is paid for by taxes and social security contribution, which stay as they are. Potential additional costs are covered by a new tax on capital.
	Increased taxes and contributions	The basic income is paid for by income taxes and social security contributions, which will increase in the future.

*The total vignette universe is 3×3×2×4×3 = 216. ** One policy design dimension, of accumulation, is held constant across the vignettes. This means that all respondents are told that *“people who work will receive the money they earn elsewhere on top of the basic income”*.

**Table 3:** Dimensions, levels and wording for the first vignette experiment on policy outcomes.

Dimensions	Levels	Vignette text
Poverty	Decrease	The poverty rate in Belgium will decrease.
	Increase	The poverty rate in Belgium will increase.
	Stagnate	The poverty rate in Belgium will remain virtually the same.
Income inequality	Decrease	The gap between the rich and poor will decrease.
	Increase	The gap between the rich and poor will increase.
	Stagnate	The gap between the rich and poor will remain virtually the same.
Unemployment	Decrease	The number of unemployed people in Belgium will decrease.
	Increase	The number of unemployed people in Belgium will increase.
	Stagnate	The number of unemployed people in Belgium will remain virtually the same.
Entrepreneurship	Decrease	The number of entrepreneurs will decrease.
	Increase	The number of entrepreneurs will increase.
	Stagnate	The number of entrepreneurs will remain virtually the same.
Informal care	Decrease	The number of informal caregivers will decrease.
	Increase	The number of informal caregivers will increase.
	Stagnate	The number of informal caregivers will remain virtually the same.

*The total vignette universe is 3×3×3×3×3 = 243. **Informal caregivers were defined in the survey as “people who provide voluntary and unpaid care to people with physical, mental or (social) psychological disabilities in their family, household or social network”.

**Table 4:** Average level of support for basic income by randomized levels.

Dimensions	Average level of support (0-100)
Policy design	Q5
Q54: Amount	
500€	52.5
1000€	54.3
1500€	57.5
Q55: Conditionality	
Not required to work	51.2
Required to care or volunteer	58.1
Q56: Universality	
Everyone including newcomers	52.9
Residency requirement	56.0
Nationality requirement	55.2
Q57: Integration within existing system	
Replace all benefits	53.8
Replace several benefits	52.7
Replaces benefits but supplements	56.7
Replaces no benefits	55.3
Q58: Financing	
Climate tax	55.8
Capital tax	56.4
Increase in taxes and contributions	51.7
Policy outcomes	Q9
Q59: Poverty	
Decrease	53.0
Increase	42.7
Stagnate	47.4
Q60: Income inequality	
Decrease	52.3
Increase	49.7
Stagnate	47.3
Q61: Unemployment	
Decrease	53.6
Increase	42.8
Stagnate	46.9
Q62: Entrepreneurship	
Decrease	44.4
Increase	49.9
Stagnate	48.8
Q63: Informal care	
Decrease	46.2
Increase	49.7
Stagnate	47.3

*The reported numbers represent the marginal means. The numbered “Q's” refer to the relevant variables in the dataset.

## 3 Experimental Design, Materials and Methods

Most of the survey items were taken from prior, well-established surveys, most notably the European Social Survey (ESS) and the Belgian National Election Study (BNES). Other items were newly designed specifically for the BABEL survey, and were therefore extensively pre-tested in cognitive interviews. The full questionnaire was also piloted among a student sample, before it was fielded among a larger sample of the Belgian population.

The questionnaire, which took on average about 20 minutes to complete in the platform “Qualtrics”, consists of five modules. The first and core module is about the survey's central topic: basic income. To set the scene, respondents are asked open questions about their familiarity with the concept. These questions were followed by a researcher-determined definition of basic income. Important to note, however, is that different respondents received different descriptions, as the design characteristics were randomly varied across respondents in a full factorial vignette experiment. In our experiment, respondents are presented with a vignette depicting a hypothetical (but realistic) basic income scheme that is randomly varied on five important policy design dimensions: amount, universality, conditionality, integration within the existing social security system and financing. After reading the vignette, respondents are asked to indicate to what extent they (1) support the introduction of that particular basic income scheme in Belgium; (2) see it as an improvement compared to the current social security system; (3) think they will personally benefit from it; and (4) believe it will yield greater social justice.

The first vignette experiment is immediately followed by a second one, which aims to investigate how support for basic income is affected by its policy outcomes. After respondents have read and rated the first vignette, they are told that the Belgian government has commissioned a scientific study on the likely consequences of introducing a basic income in Belgium. They are then shown a second vignette describing the (hypothetical) results of this study. More specifically, the vignette randomly varies the effects a basic income would produce in terms of poverty, income inequality, unemployment, entrepreneurship and informal care. After reading the vignette, respondents are again asked to indicate their support for introducing basic income in Belgium, but only this time bearing the knowledge on its likely policy outcomes in mind. Respondents are informed about the hypothetical (and deceptive) nature of the presented research results at the end of the survey, in the debriefing.

In addition to the vignettes, the basic income module includes items tapping into support for three so-called ‘cousins’ of basic income: a negative income tax [2], a one-off endowment or stakeholder grant [3] and a basic income that is restricted to a particular age category, benefitting only young adults, the working-age population or pensioners [4]. The module also asks respondents how they would react in case a basic income was actually introduced in Belgium. Would they work less, stop working completely, or find a new job? Would they do more volunteering or caring? Would they start their own business or start studying again? Or would they change nothing at all? These questions about self-predicted reactions to a basic income were included to shed light on its behavioral feasibility [5].

The second module deals with attitudes and experiences regarding existing social security, which were included because they are likely to influence people's stance towards basic income [6]. Respondents were asked about: (1) their overall trust in the current social security system; (2) their perceptions of the economic, moral and social consequences of that system; and (3) their support for cutting back specific schemes, including pensions, unemployment insurance, sickness and disability benefits, social assistance, and child allowances. The survey also probes respondents’ receipt of these benefits (either personally, in their household or in their broader network of family and friends) in the past 12 months, as well as the predicted likelihood of receiving them in the coming 12 months. The latter was, however, not asked for pensions and child allowances, given the predictable nature of the social risks they address.

The third module assesses a number of sociodemographic characteristics which will structure support for basic income (van Oorschot & Roosma, 2020). More specifically, these characteristics are: (1) region of residence, (2) work status, (3) social statute (e.g. private or public sector employee), (4) employment contract, (5) highest attained degree, (6) monthly net household income and subjective evaluations thereof, (7) household composition, and (8) country of birth. The latter also includes the country of birth of respondents’ parents, so that both first-generation and second-generation migrants can be identified in the sample.

The fourth module measures some political beliefs and affiliations that are likely to shape support for basic income [7]. Besides a standard left-right self-placement scale, respondents were also asked about voting intentions, as if it were federal elections on the day the survey was completed. In practice, respondents could either select a political party from a list (which varies across regions because of the specific Belgian electoral system), or they could indicate that they would cast an invalid vote or not vote at all (even though this is compulsory in Belgium). Respondents were also asked whether they are (or were) member of a political party and a trade union, and if so, which one. With regard to the latter, it is worth repeating that trade union members were deliberately oversampled in our study, because of the prominent role trade unions continue to play in the Belgian welfare state.

The fifth and final module taps into respondents’ experiences with the Covid-19 pandemic. Respondents were asked: (1) whether they (or someone else in their household) have been infected by the Covid-19 virus; (2) what has happened to their standard of living since the outbreak of the pandemic; and (3) whether the pandemic has led to negative life events such as dismissal, temporary unemployment, or psychological problems.

## Ethics Statements

Participation in the survey was voluntary and respondents were informed that their data would be processed anonymously.

## CRediT authorship contribution statement

**Tijs Laenen:** Conceptualization, Methodology, Writing – review & editing. **Marie-Laure Mulayi:** Conceptualization, Writing – original draft, Writing – review & editing. **Cyrille Francisco:** Writing – review & editing. **Wim Van Lancker:** Writing – review & editing, Supervision.

## Declaration of Competing Interest

The authors declare that they have no known competing financial interests or personal relationships that could have appeared to influence the work reported in this paper.

## Data Availability

Babel survey (Original data) (OSF).

## Appendix Supplementary materials

Image, application 1

Image, application 2
