# Supplementary material for: Basic Income in Belgium survey: experimental data on citizens’ attitudes toward a variety of basic income policies
Source: Data Brief. 2023 Jul 7;49:109376. doi: 10.1016/j.dib.2023.109376 (PMC10368754; doi:10.1016/j.dib.2023.109376)
Supplement: Supplementary file 2 [file mmc2.docx]

**Annex**

**QUESTIONNAIRE BABEL SURVEY**

**RESEARCH ON THE PERCEPTION AND OPINION OF BELGIANS TOWARD A UNIVERSAL BASIC INCOME**

- **Basic Income**

**2021**

**In some countries, there is a debate on whether or not to introduce a basic income. A number of questions about the introduction of a basic income in Belgium are presented below.**

**A1. Have you ever heard of the term "basic income"?**

- Yes
- No

If A1 is ‘yes’, then A2 and A3. If A1 is ‘no’, then A4.

**A2. Through which channel did you hear of a basic income? Multiple answers are possible.**

- The media: television, newspapers, etc.
- Social networks: Twitter, Facebook, etc.
- Friends and family
- Colleagues
- School/University/High School
- Other:………………………………………………………………………….

**A3. How would you describe in your own words what a "basic income" is?**

………………………………………………………………………………………………..

**A4. What do you think a “basic income” is?**

………………………………………………………………………………………………..

**A5. VIGNETTE 1. Let us assume that the Belgian government wants to introduce a basic income. This basic income would have the following characteristics:**

- The Belgian government provides a monthly income of €500/€1000/€1500.
- That amount is paid to *all adult residents in Belgium, including newcoming migrants/ all adults living in Belgium, on the condition that they been in the country for a few years/ all adults living in Belgium, on the condition that they have the Belgian nationality.*
- Working people receive the money they earn themselves in addition to the Basic Income.
- People who are not working are *not obliged to search for a paid job/ obliged to do voluntary work or to take up caring responsibilities.*
- The Basic income *replaces all existing social benefits/ replaces some existing social benefits, such as child allowances and sickness benefits/ replaces some existing social benefits but provides top-ups for people with additional needs or costs (e.g. people with children or disabled people) / replaces no existing social benefits.*
- The basic income is paid for by income taxes and social security contribution, which *stay as they are. Potential additional costs are covered by a new climate tax on CO2 emissions/ stay as they are. Potential additional costs are covered by a new tax on capital/ will increase in the future.*

***A5.1. To what extent are you in favor or against the introduction of this basic income in Belgium?***

Against For

| **0** | **1** | **2** | **3** | **4** | **5** | **6** | **7** | **8** | **9** | **10** |
| --- | --- | --- | --- | --- | --- | --- | --- | --- | --- | --- |

***A5.2. To what extent do you see this basic income as a deterioration or an amelioration of current social security?***

Detoriate Improve

| **0** | **1** | **2** | **3** | **4** | **5** | **6** | **7** | **8** | **9** | **10** |
| --- | --- | --- | --- | --- | --- | --- | --- | --- | --- | --- |

***A5.3. To what extent do you think you will personally benefit from this basic income?***

No personal benefit A lot of personal benefit

| **0** | **1** | **2** | **3** | **4** | **5** | **6** | **7** | **8** | **9** | **10** |
| --- | --- | --- | --- | --- | --- | --- | --- | --- | --- | --- |

***A5.4. To what extent do you think this basic income will create more or less justice in our society?***

| **0** | **1** | **2** | **3** | **4** | **5** | **6** | **7** | **8** | **9** | **10** |
| --- | --- | --- | --- | --- | --- | --- | --- | --- | --- | --- |

Less Just More just

**A6. VIGNETTE 2. Afin d’estimer l’impact de l’introduction d’un revenu de base sur la société Belge, le gouvernement a fait réaliser une étude par plusieurs universités Belges. Cette étude montre que :**

Informal caregivers were defined in the survey as “people who provide voluntary and unpaid care to people with physical, mental or (social) psychological disabilities in their family, household or social network”.1

- The poverty rate in Belgium decrease/increase/ remain virtually the same.
- The gap between the rich and poor will decrease/increase/remain virtually the same.
- The number of unemployed people in Belgium will decrease/increase/remain virtually the same.
- The number of entrepreneurs will decrease/increase/remain virtually the same.
- The number of informal caregivers will decrease/increase/remain virtually the same.

***A6.1. Considering this information, we would like to ask you again to what extent are you for or against the introduction of a basic income in Belgium?***

Against For

| **0** | **1** | **2** | **3** | **4** | **5** | **6** | **7** | **8** | **9** | **10** |
| --- | --- | --- | --- | --- | --- | --- | --- | --- | --- | --- |

***A6.2. To what extent do you see this basic income as improving or worsening current social security?***

Detoriate Improve

| **0** | **1** | **2** | **3** | **4** | **5** | **6** | **7** | **8** | **9** | **10** |
| --- | --- | --- | --- | --- | --- | --- | --- | --- | --- | --- |

***A6.3. To what extent do you trust the results of this scientific research?***

Low trust High trust

| **0** | **1** | **2** | **3** | **4** | **5** | **6** | **7** | **8** | **9** | **10** |
| --- | --- | --- | --- | --- | --- | --- | --- | --- | --- | --- |

**A7. Some people are proposing a negative income tax instead of a basic income. That is a system where below a certain income threshold you get a basic income from taxes. As your income increases that basic income decreases. When your income rises back above the income threshold you start paying taxes again.**

***To what extent are you in favor or against the introduction of such negative income tax in Belgium?***

Against For

| **0** | **1** | **2** | **3** | **4** | **5** | **6** | **7** | **8** | **9** | **10** |
| --- | --- | --- | --- | --- | --- | --- | --- | --- | --- | --- |

**A8. Some say that a basic income could also be paid once instead of monthly. Each citizen would then receive a basic endowment from the government once in his or her lifetime. People are free to choose how to spend that money.**

***To what extent are you in favor or against the introduction of such basic endowment in Belgium?***

Against For

| **0** | **1** | **2** | **3** | **4** | **5** | **6** | **7** | **8** | **9** | **10** |
| --- | --- | --- | --- | --- | --- | --- | --- | --- | --- | --- |

**A9. Finally, a basic income can be limited to certain age groups.**

***A9.1. To what extent are you in favor or against the introduction of a basic income only for people over the age of 65?***

Against For

| **0** | **1** | **2** | **3** | **4** | **5** | **6** | **7** | **8** | **9** | **10** |
| --- | --- | --- | --- | --- | --- | --- | --- | --- | --- | --- |

***A9.2. To what extent are you in favor or against the introduction of a basic income only for people of working age, between 18 and 65?***

Against For

| **0** | **1** | **2** | **3** | **4** | **5** | **6** | **7** | **8** | **9** | **10** |
| --- | --- | --- | --- | --- | --- | --- | --- | --- | --- | --- |

***A9.3. To what extent are you in favor or against the introduction of a basic income only for young people between the ages of 18 and 25?***

Against For

| **0** | **1** | **2** | **3** | **4** | **5** | **6** | **7** | **8** | **9** | **10** |
| --- | --- | --- | --- | --- | --- | --- | --- | --- | --- | --- |

**A10. Now we would like to know what you would do if you received a monthly basic**

**income from the government. Multiple answers are possible.**

- I would quit work completely
- I would reduce my working hours
- I would vonlunteer
- I would start a business
- I would provide more care for others
- I would look for a new job
- I would follow a training course
- I would not change anything
- **Attitudes towards social security**

**Here are some questions about Belgian social security. Our social security compensates people for loss of income due to social risks such as ageing, illness and unemployment.**

**B1. To what extent do you trust the Belgian social security system?**

Little trust High trust

| **0** | **1** | **2** | **3** | **4** | **5** | **6** | **7** | **8** | **9** | **10** |
| --- | --- | --- | --- | --- | --- | --- | --- | --- | --- | --- |

**B2. To what extent are you in favor or against budget cuts in the following social benefits?**

- ***Retirement pension (from 65 years old)***

Against For

| **0** | **1** | **2** | **3** | **4** | **5** | **6** | **7** | **8** | **9** | **10** |
| --- | --- | --- | --- | --- | --- | --- | --- | --- | --- | --- |

- ***Early retirement***

Against For

| **0** | **1** | **2** | **3** | **4** | **5** | **6** | **7** | **8** | **9** | **10** |
| --- | --- | --- | --- | --- | --- | --- | --- | --- | --- | --- |

- ***Unemployment benefit***

Against For

| **0** | **1** | **2** | **3** | **4** | **5** | **6** | **7** | **8** | **9** | **10** |
| --- | --- | --- | --- | --- | --- | --- | --- | --- | --- | --- |

- ***Sickness benefit***

Against For

| **0** | **1** | **2** | **3** | **4** | **5** | **6** | **7** | **8** | **9** | **10** |
| --- | --- | --- | --- | --- | --- | --- | --- | --- | --- | --- |

- ***Assistance benefit***

Against For

| **0** | **1** | **2** | **3** | **4** | **5** | **6** | **7** | **8** | **9** | **10** |
| --- | --- | --- | --- | --- | --- | --- | --- | --- | --- | --- |

- ***Child benefit***

Against For

| **0** | **1** | **2** | **3** | **4** | **5** | **6** | **7** | **8** | **9** | **10** |
| --- | --- | --- | --- | --- | --- | --- | --- | --- | --- | --- |

**B3. To what extent do you agree or disagree with the following statements?**

|  |  | Completely disagree | Disagree | Neither agree nor disagree | Agree | Completely agree | I don’t know |
| --- | --- | --- | --- | --- | --- | --- | --- |
| 1. | Our social security costs businesses too much and harms our economy | 1 | 2 | 3 | 4 | 5 | 6 |
| 2. | Our social security demotivates people to work. | 1 | 2 | 3 | 4 | 5 | 6 |
| 3. | Our social security prevents widespread poverty. | 1 | 2 | 3 | 4 | 5 | 6 |
| 4. | Our social security make people less willing to care for one another. | 1 | 2 | 3 | 4 | 5 | 6 |
| 5. | Our social security attracts to many migrants. | 1 | 2 | 3 | 4 | 5 | 6 |

- **Benefit recipiency**

**C1. In the past 12 months, which of the following social security benefits have you personally received? Multiple answers are possible.**

- Retirement pension
- Early retirement pension
- Unemployment benefit
- Sickness benefit
- Assistance benefit
- Child benefit
- Other:…………………………………………………………………………
- None

**C2. In the past 12 months, which of the following social security benefits has someone else from your household received? Multiple answers are possible.**

- Retirement pension
- Early retirement pension
- Unemployment benefit
- Sickness benefit
- Assistance benefit
- Child benefit
- other:…………………………………………………………………………
- None

**C3. In the past 12 months, which of the following social security benefits has at least one person in your family or circle of friends received? Multiple answers are possible.**

- Retirement pension
- Early retirement pension
- Unemployment benefit
- Sickness benefit
- Assistance benefit
- Child benefit
- Other:…………………………………………………………………………
- None

**C4. How likely do you think that you will receive the following social security benefits in the next 12 months?**

- ***Unemployment benefit***

Very low Very high

| 0 | 1 | 2 | 3 | 4 | 5 | 6 | 7 | 8 | 9 | 10 |
| --- | --- | --- | --- | --- | --- | --- | --- | --- | --- | --- |

- ***Sickness benefit***

Very low Very high

| 0 | 1 | 2 | 3 | 4 | 5 | 6 | 7 | 8 | 9 | 10 |
| --- | --- | --- | --- | --- | --- | --- | --- | --- | --- | --- |

- ***Social assistance benefit***

Very low Very high

| 0 | 1 | 2 | 3 | 4 | 5 | 6 | 7 | 8 | 9 | 10 |
| --- | --- | --- | --- | --- | --- | --- | --- | --- | --- | --- |

- **Demographic information**

**D1. In which region do you live? Indicate only your main residence.**

- Flanders
- Wallonia
- Brussels

**D2. Do you currently have a paid job?**

- Yes
- No

If ‘no’ on D2, then D3 & D4. If ‘yes’ on D2, then D5.

**D3. Which description best fits your current situation?**

- Retired
- On sick leave or disabled
- Housewife/man
- Pursuing full-time education
- Unemployed and looking for work
- Unemployed and not looking for work
- Doing informal care
- Other:…………………………………………

**D4. Have you ever had a paid job?**

- Yes
- No

If ‘yes’ on D4, then D5.

**D5. What is your current employment type?**

1. Blue-collar worker

2. White-collar worker

3. Self-employed

4. Helper or self-employed

5. Freelancer

6. Student

7. Civil Servant

8. Other:…………………………………………….

**D6. What type of employment contract do you currently have?**

1. Full-time, permanent contract

2. Full-time, temporary contract

3. Part-time, permanent contract

4. Part-time, temporary contract

5. Other:……………………………………………...

**D7. What is your highest obtained degree?**

1. None

2. Primary education

3. Lower secondary education: vocational

4. Lower secondary education: technical

5. Lower secondary education: general

6. Higher secondary education: vocational

7. Higher secondary education: technical

8. Higher secondary education: general

9. Non-university degree

10. University degree

11. Other:………………………………………………………………

**D8. Could you indicate in which category the net income of your household generally falls?**

1. Less than 500 €

2. 501 to 1000 €

3. 1001 to 1500 €

4. 1501 to 2000 €

5. 2001 to 2500 €

6. 2501 to 3000 €

7. 3001 to 3500 €

8. 3501 to 4000 €

9. 4001 to 4500 €

10. 4501 to 5000 €

11. 5001 to 5500 €

12. 5501 to 6000 €

13. 6001 to 6500 €

14. 6501 to 7000 €

15. 7001 to 7500 €

16. 7501 to 8000 €

17. Higher than 8000 €

**D9. How many adults, yourself included, depend on this income?**

………………… [number] adults

**D10. How many underaged children are dependent on this income?**

………………… [number] children

If number of children is ≥ 1, then A11.

**D11. How many children in your household are under the age of 14yo?**

………………… [number]children

**D12. Some people have a good income, others have to make do with less. Which of the following statements best describes your current situation, taking into account the total income that you currently have?**

- We have more than enough, we can easily save money
- We have enough to get along, without Difficulties
- We have just enough to get along
- We do not have enough and often have difficulties to get along

**D13. Please try to remember your standard of living five years ago. Compared to your current situation, do you feel things have gotten worse, have gotten better, or stayed the same?**

- Got much worse
- Got worse
- Stayed about the same
- Got better
- Got much better

**D14. Are you currently a member of a trade union ?**

- Yes
- No

If D14 is ‘no’, then D15. If D14 is ‘yes’, then D16.

**D15. Have you ever been a member or a trade union?**

- Yes
- No

**D16. Of which trade union are you a member?**

- ABVV
- ACV
- ACLVB
- FGTB
- CSC
- CGSLB
- Other:………..................................

**D17. In which country were you born?**

1. Belgium

2. Other:…………………………………………

3. Don’t know

**D18. In which country was your father born?**

1. Belgium

2. Other:…………………………………………

3. Don’t know

**D19. In which country was your mother born?**

1. Belgium

2. Other:…………………………………………

3. Don’t know

- **Political orientation**

**E1. In politics people sometimes talk of “left” and “right”. Where would you place yourself on this scale, where 0 means “left” and 100 means “right”?**

All the way to the left All the way to the right

| 0 | 1 | 2 | 3 | 4 | 5 | 6 | 7 | 8 | 9 | 10 |
| --- | --- | --- | --- | --- | --- | --- | --- | --- | --- | --- |

**E2. Which political party would you vote for should the federal elections be held today?**

If ‘1’ on D1, then E2.

| cdH | 1 |
| --- | --- |
| ECOLO | 2 |
| MR! | 3 |
| PVDA-PTB | 4 |
| PS | 5 |
| Défi | 6 |
| Other party | 7 |
| Blank | 8 |
| Invalid | 9 |
| Would not vote | 10 |

**E3. Which political party would you vote for should the federal elections be held today?**

| cdH | 1 |
| --- | --- |
| Groen | 2 |
| N-VA | 3 |
| Open VLD | 4 |
| PVDA-PTB | 5 |
| Vlaams Belang | 6 |
| Other party | 7 |
| Blank | 8 |
| Invalid | 9 |
| Would not vote | 10 |

If ‘2’ on D1, then E2.

**E4. . Which political party would you vote for should the federal elections be held today?**

| CD&V | 1 |
| --- | --- |
| cdH | 2 |
| Groen | 3 |
| ECOLO | 4 |
| N-VA | 5 |
| Open VLD | 6 |
| MR! | 7 |
| PVDA-PTB | 8 |
| One-Brussels.VOORUIT(Sp.a) | 9 |
| PS | 10 |
| Vlaams Belang | 11 |
| Défi | 12 |
| Other party | 13 |
| Blank | 14 |
| Invalid | 15 |
| Would not vote | 16 |

If ‘3’ on D1, then E3.

**E5. Are you currently a member of a political party?**

- Yes
- No

If E4 is ‘yes’, then E5 or E6. If E4 is ‘no’, then F1.

**E6. Which political party are you currently a member of?**

If ‘1’ on D1, then E5.

| cdH | 1 |
| --- | --- |
| ECOLO | 2 |
| MR! | 3 |
| PVDA-PTB | 4 |
| PS | 5 |
| Défi | 6 |
| Other party | 7 |
| Blank | 8 |
| Invalid | 9 |
| Would not vote | 10 |

**E7. Which political party are you currently a member of?**

If ‘2’ on D1, then E5.

| cdH | 1 |
| --- | --- |
| Groen | 2 |
| N-VA | 3 |
| Open VLD | 4 |
| PVDA-PTB | 5 |
| Vlaams Belang | 6 |
| Other party | 7 |
| Blank | 8 |
| Invalid | 9 |
| Would not vote | 10 |

**E8 Which political party are you currently a member of?**

If ‘3’ on D1, then E6.

| CD&V | 1 |
| --- | --- |
| cdH | 2 |
| Groen | 3 |
| ECOLO | 4 |
| N-VA | 5 |
| Open VLD | 6 |
| MR! | 7 |
| PVDA-PTB | 8 |
| One-Brussels.VOORUIT(Sp.a) | 9 |
| PS | 10 |
| Vlaams Belang | 11 |
| Défi | 12 |
| Other party | 13 |
| Blank | 14 |
| Invalid | 15 |
| Would not vote | 16 |

- **COVID-19**

**F1. Have you been infected with Covid-19?**

- Yes, I tested positive for Covid-19
- Yes, I think I have had Covid-19 but I’ve not been tested
- I did not have Covid-19

**F2. Has anyone else in your household been infected with the coronavirus?**

- Yes, someone else from my household has tested positive for Covid-19
- I think someone else from my household had Covid-19, but that person has not been tested
- No, no other person from my household has had Covid-19

**F3. Can you tell me if the following situations have occurred in your life as a result of the Covid-19 pandemic? Indicate all situations that apply to you now or in the past.**

- I have been fired
- I have been temporarily unemployed
- I have been put in quarantined automatically or on vonluntary basis
- I have been hospitatilised due to COVID-19
- I have financial troubles due to COVID-19
- I have psycological problems due to COVID-19
- I had to close my business
- None of those situations

**F4. Please try to remember how you were before the outbreak of covid-19, in terms of your standard of living. Compared to your current situation, do you feel things have gotten worse, have gotten better, or stayed the same?**

- Got much worse
- Got worse
- Stayed about the same
- Got better
- Got much better

- **Debriefing**

You have been informed in advance about the possible social consequences of the introduction of a basic income in Belgium. However, the consequences presented to you are fictitious. Therefore, for the sake of clarity, they are not the result of a study carried out by several Belgian universities. The possible consequences of the introduction of a basic income in Belgium are currently being analyzed by researchers from the KU Leuven, the University of Antwerp and the University of Saint Louis-Brussels.
